# Needs and resources of people with type 2 diabetes in peri-urban Cochabamba, Bolivia: a people-centred perspective

**DOI:** 10.1186/s12939-021-01442-1

**Published:** 2021-04-20

**Authors:** Christine Cécile Leyns, Niek Couvreur, Sara Willems, Ann Van Hecke

**Affiliations:** 1 Fundación Vida Plena, Juan Capriles 346, Cochabamba, Bolivia; 2 Department of Public Health and Primary Care, Faculty of Medicine and Health Sciences, Ghent University, Ghent, Belgium; 3 University Center for Nursing and Midwifery, Ghent University Hospital, Ghent, Belgium

**Keywords:** Patient-Centred care, Bolivia, Diabetes mellitus, Long-term care, Health literacy, Community participation, Developing countries, Self-management, Health planning

## Abstract

**Background:**

The rising prevalence of type 2 diabetes results in a worldwide public healthcare crisis, especially in low- and middle-income countries (LMICs) with unprepared and overburdened health systems mainly focused on infectious diseases and maternal and child health. Studies regarding type 2 diabetes in LMICs describe specific interventions ignoring a comprehensive analysis of the local factors people see influential to their health. This study aims to meet this research gap by exploring what people with type 2 diabetes in Bolivia need to maintain or improve their health, how important they perceive those identified needs and to what extent these needs are met.

**Methods:**

From March until May 2019, 33 persons with type 2 diabetes from three periurban municipalities of the department of Cochabamba participated in this study. The concept mapping methodology by Trochim, a highly structured qualitative brainstorming method, was used to generate and structure a broad range of perspectives on what the participants considered instrumental for their health.

**Results:**

The brainstorming resulted in 156 original statements condensed into 72 conceptually different needs and resources, structured under nine conceptual clusters and four action domains. These domains illustrated with vital needs were: (1) self-management with use of plants and the possibility to measure sugar levels periodically; (2) healthcare providers with the need to trust and receive a uniform diagnosis and treatment plan; (3) health system with opportune access to care and (4) community with community participation in health and safety, including removal of stray dogs.

**Conclusions:**

This study identifies mostly contextual factors like low literacy levels, linguistic problems in care, the need to articulate people’s worldview including traditional use of natural remedies with the Bolivian health system and the lack of expertise on type 2 diabetes by primary health care providers. Understanding the needs and structuring them in different areas wherein action is required serves as a foundation for the planning and evaluation of an integrated people centred care program for people with type 2 diabetes. This participative method serves as a tool to implement the often theoretical concept of integrated people centred health care in health policy and program development.

## RESUMEN

**Antecedentes:**

La creciente prevalencia de la diabetes tipo 2 resulta en una crisis mundial de salud pública, especialmente en países de ingresos bajos y medianos (PIBM) con sistemas de salud no preparados y sobrecargados, centrados principalmente en las enfermedades infecciosas y la salud materno infantil. Los estudios sobre la diabetes tipo 2 en los PIBM describen intervenciones específicas ignorando un análisis exhaustivo de los factores locales que las personas consideran influyen en su salud. Este estudio tiene como objetivo cubrir esta brecha de investigación al explorar lo que las personas con diabetes tipo 2 en Bolivia necesitan para mantener o mejorar su salud, qué tan importante perciben esas necesidades identificadas y en qué medida estas necesidades son satisfechas.

**Métodos:**

De marzo a mayo de 2019, 33 personas con diabetes tipo 2 de tres municipios periurbanos del departamento de Cochabamba participaron en este estudio. Se utilizó la metodología de mapeo conceptual de Trochim, un método de lluvia de ideas cualitativo altamente estructurado, para generar y estructurar una amplia gama de perspectivas sobre lo que los participantes consideraron herramientas para su salud.

**Resultados:**

La lluvia de ideas resultó en 156 enunciaciones originales condensadas en 72 necesidades y recursos conceptualmente diferentes, estructurados en nueve grupos conceptuales y cuatro dominios de acción. Estos dominios ilustrados con necesidades vitales fueron: (1) autogestión con el uso de plantas y la posibilidad de medir los niveles de azúcar periódicamente; (2) proveedores de salud con la necesidad de confiar y recibir un diagnóstico y plan de tratamiento uniforme; (3) sistema de salud con acceso oportuno a la atención y (4) comunidad con participación comunitaria en salud y seguridad, incluida el control de perros callejeros.

**Conclusiones:**

Este estudio identifica principalmente factores contextuales como bajos niveles de alfabetización, problemas lingüísticos en la atención, la necesidad de articular la cosmovisión de las personas incluyendo el uso tradicional de remedios naturales con el sistema de salud boliviano y la falta de pericia en diabetes tipo 2 por parte de los proveedores de atención primaria de salud. Comprender las necesidades y estructurarlas en diferentes áreas en las que se requiere actuar, sirve como base para la planificación y evaluación de un programa de atención integral centrada en la persona para personas con diabetes tipo 2. Este método participativo sirve como una herramienta para implementar el concepto, a menudo teórico, de atención integrada centrada en las personas en el desarollo de políticas y programas de salud.

## Background

The prevalence of diabetes is estimated to increase from 415 million in 2015 to 642 million by 2040, resulting in a public healthcare crisis worldwide [1]. The steep increase of primarily type 2 diabetes (T2D) can be attributed to urbanisation, changing diets and decreasing physical activity [2, 3]. Untreated T2D has serious physical, psychological and social consequences [4, 5]. Individuals, families and communities suffer financial hardship [4] and health systems are put under pressure [6].

Diabetes disproportionally affects low- and middle-income countries (LMICs). More than 70% of cases [7] and 80% of deaths caused by diabetes [5] are expected to take place in LMICs by 2030. Health systems based on comprehensive and community oriented primary health care (PHC) are better equipped to face the growing burden of T2D [8]. However, PHC lacks comprehensiveness in most LMICs where it is focused on episodic treatment, especially of infectious disease and maternal and child health [9]. Concurrently, the lion’s share of evidence on comprehensive diabetes programs in PHC comes from high-income countries [10], making it less pertinent for LMICs [11, 12].

Previous studies on improving diabetes management at the PHC level in LMICs were identified with following Boolean operators: (“community health service*” OR “rural health service*” OR “community health centre*” OR “community health nursing” OR “Primary health centre” OR “primary health care centre”) AND “diabetes”, including studies in LMICs on management of T2D published until December 2019. The 23 studies, from 15 different LMICs, found, mainly focused on isolated interventions like the training of formal or informal healthcare providers [12–15], pharmacological follow-up [16] or patient education and counselling [14, 17–22]. Despite their seemingly positive short-term effects, managing chronic conditions requires a comprehensive strategy which involves more than performing a series of disconnected interventions [23, 24]. To identify what can work within the local context, and to promote community ownership, engaging individuals with T2D, their families and communities in the design and implementation of prevention and treatment solutions are to be considered [25–30]. This type of healthcare design is known as ‘People-Centred Health Care’, defined as ‘an approach to care that consciously adopts individuals’, caregivers’, families’ and communities’ perspectives as participants in, and beneficiaries of, trusted health systems that respond to their needs and preferences in humane and holistic ways’ [31]. No studies were found that thoroughly explored the needs and resources of people living with T2D in Bolivia.

Bolivia is a LMIC with a human development index of 0.70 in 2018, ranked as 15th of the 20 countries in Latin-America [32]. It is characterised by a high prevalence of chronic disease and a weak PHC system [33] with a long history of selective programs and a biomedical hospital centric focus [34]. Notwithstanding the introduction in 2008 [35] of a public health care model based on intercultural community family health, encouraging broad participation while incorporating both Western and indigenous (traditional) medicines, progress has not been evaluated [36, 37]. The prevalence of T2D in the main urban regions was estimated at around 7.2% in 2001, with no later studies on country level prevalence available [38]. The department of Cochabamba has tropical lowlands, semi-arid valleys and highlands. This study was performed in Sacaba (172,466 inhabitants) and Quillacollo (137,182 inhabitants), the second and third largest municipalities situated at both sides of the urbanised valley of Cochabamba. They are the fastest growing municipalities with population growths of respectively 150 and 100% since 1992, reaching a population density of 83.14 and 59.7 inhabitants/km2 and poverty levels of 36.3% (72.56% in 1992) and 28.3% (62.88% in 1992) [39]. In both municipalities, over 60% of the population identified themselves as Quechua, received a median of 8 years of schooling with 10% illiteracy [40]. While traditionally working in agriculture [40, 41], they now predominantly work in transport and trade. In Quillacollo and Sacaba respectively, 50.7 and 63.5% of the population access the public health system, 29.2 and 22.8% access private health services, 23 and 18.7% access a social health insurance service, 10.4 and 12.5% consult a traditional healer while half of the population uses home remedies or self-medicates [39]. To be able to design an inclusive, efficient and acceptable diabetes program for people living with T2D, it is necessary to grasp their current resources, how these can be bolstered and which needs are unmet. The aim of this study was threefold (1) to obtain an extensive set of perspectives on what people who live with T2D in the recently urbanised region of Cochabamba, Bolivia rely on, (2) to explore what they need to maintain their health, and (3) to structure these elements in a people-centred diabetes care plan.

## Methods

The concept mapping methodology by Trochim [42], a highly structured qualitative group method for brainstorming and idea sharing, was selected for its ability to create a comprehensive framework for evaluation and planning of diabetes management in the communities under study.

### Participants

A total of 33 people with T2D were recruited from March until May 2019 in Cochabamba, Bolivia: 14 from Sacaba, 16 from Quillacollo and 3 from the highland municipality of Punata (28,887 inhabitants). A convenience sample was used. All participants were over 18, had T2D and spoke Spanish as a first or second language. Fourteen people took part in the generation of statements related to needs and resources as well as in the valorisation of these statements. Nineteen additional participants took part in the rating phase, resulting in 33 people participating in this study.

#### Recruitment procedure

The greater the variety of the participants, the richer the outcome of the concept map. People with diverse socio-economic backgrounds were selected to participate in this study through different recruitment strategies. First, nine participants were recruited by joining outreaches of a local primary health care centre in the peri-urban part of Sacaba. These participants were known by the public healthcare centre and were asked to participate by their healthcare providers. Second, the researcher encountered five additional participants in the same peri-urban zone through direct community contact. These latter participants were formerly diagnosed with T2D, but unknown by the local public health centre. Only these 14 participants took part in the statement generation phase. The remaining 19 participants were recruited by joining an outreach of a general practitioner working in the peri-urban part of the municipality of Quillacollo, and in the rural municipality of Punata. The general practitioner asked these 19 persons to participate in this study. All participants were diagnosed with T2D prior to this study, and signed an informed consent.

### Data collection

#### Design

**Fig. 1:**
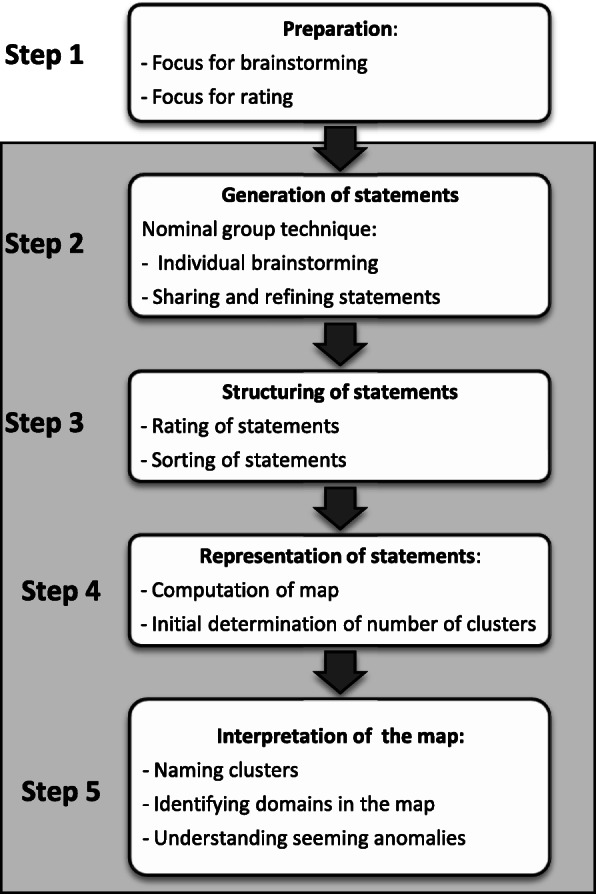
The Concept Mapping Process (Adapted from Leyns et al., 2018) [27]

The concept mapping methodology by Trochim [42] is well suited to deal with complex human systems in public health [43]. It is inductive, based on major ideas of participants that are interrelated to design a well-structured concept map which provides feedback to the system under study. The methodology consists of a multi-step process as seen in Fig. 1. Firstly, the focus of brainstorming and ratings is defined. Secondly, participants generate a set of statements during a brainstorming session. Thirdly, these statements are structured during a rating and sorting process and fourthly they are represented in a concept map, visualising the statements, their ratings and how they are interrelated [42].

##### Step 1: preparation

The ‘seeding question’, or focus of the brainstorming, was constructed aiming for an optimal depth and breadth of desired data. This focus must be comprehensible and unambiguous for all participants. The seeding question in this study was developed by the project team and validated linguistically and culturally by a general practitioner and the group of eight people (seven of indigenous background, five illiterate) with T2D from Sacaba, who participated in the pilot concept mapping group session, resulting in: ‘Thinking as broadly as possible, what is needed to maintain or improve your health or the health of other people with diabetes in your community?’. Subsequently, the rating focus was defined, considering the kind of information desired. A Likert-scale from one to five was selected to assess the ‘importance’ and ‘presence’ of each statement [43] with the following answer categories to assess the ‘importance’: 1 (=1.00–1.49) = Not important, 2 (=1.50–2.49) = Preferable but not important, 3 (=2.50–3.49) = Important, 4 (=3.50–4.49) = Very important and 5 (=4.50–5.00) = Essential (success is very unlikely without this); to assess ‘presence’: 1 = not/never present, 2 = rarely present, 3 = sometimes present, 4 = mostly present, 5 = Very/always present. Apart from the seeding question, the selection of participants was a fundamental step in the concept mapping.

##### Step 2: generation of statements

During the first concept mapping group session participants were asked individually to think of statements in response to the abovementioned seeding question. They shared their statements one by one within a nominal group process, guaranteeing maximum equality of input [44]. Each statement was rephrased where necessary to improve understanding but was accepted without discussing its validity or relevance for other participants [43]. Very few statements, a total of 25 of which eight related to healthy food and drink intake, were generated. The project team attributed this to culturally imbedded apprehension of the participants to express themselves and low literacy levels. Five of the eight participants were assisted by a family member or a volunteer to write down statements before sharing them. The shared statements were written down and projected by a facilitator on a screen. Only three participants were able to read the projected statements while barely one completed unassisted the rating of the acquired statements with the Likert-scale. Therefore, the method of data collection and rating was adapted and respondents, including the participants of the group session, were visited individually at home. The first 14 home visits, performed in the municipality of Sacaba, started with a short discussion of the seeding question, giving the participant the possibility to reach full understanding. The participant was encouraged to think broadly in order to generate as many statements as they could think of in response to the seeding question. After generation of their own statements, statements obtained at previous home visits were read aloud and the person was asked to formulate the statements in their own words to test for understanding and reformulate the statement when necessary. Hundred fifty-six semantically different statements were formulated. These were analysed and reduced to 72 different conceptual ideas by two independent researchers.

##### Step 3: statement structuring

The importance, presence and sense of interconnectedness of the generated statements was determined through a rating and sorting process [45]. Regarding the rating, the 14 participants who generated the statements were visited a second time to perform the rating of each statement on importance (1–5) and presence (1–5) with the following questions: “How important is this need for you or other people with diabetes?” (Importance) and” Is this need fulfilled for you and for other people with diabetes?” (Presence). Nineteen additional people with T2D participated in this rating-process. Based on the experience during the pilot concept mapping group session, the ratings were done verbally, assisted by a visual representation of the rating scores. The sorting was performed by a registered nurse (researcher), a general practitioner (researcher), a political scientist and a social worker. They received the 72 statements on individual numbered cards and grouped them into piles ‘in a way that made sense to them’ [43]. Each person in the sorting process grouped the cards several times, in several ways that made sense to them.

#### Analysis

##### Step 4: representation of the statements

The 72 statements, including their rating- and sorting-data were uploaded to the free open-source concept mapping software, implemented in R [45]. Average rating scores on importance and presence for each individual statement were calculated. Subsequently, the software applied a sequence of algorithms to the sorting data, starting with the construction of a square binary matrix for each sorting task, with as many rows and columns as there were statements, to identify which statements were sorted together in piles [43]. All these matrices were added up to obtain a combined group similarity matrix. A high value in this matrix meant a high level of conceptual similarity between two statements. This resulted in a concept map with 72 different dots (in a point map), representing the statements, and 10 ‘groups of dots’, or clusters (in a cluster map). Average rating scores on importance and presence were computed for each cluster based on the ratings of the statements within. This process allowed the identification of major ideas and concepts and their interrelatedness [42].

##### Step 5: interpretation of the map

**Fig. 2:**
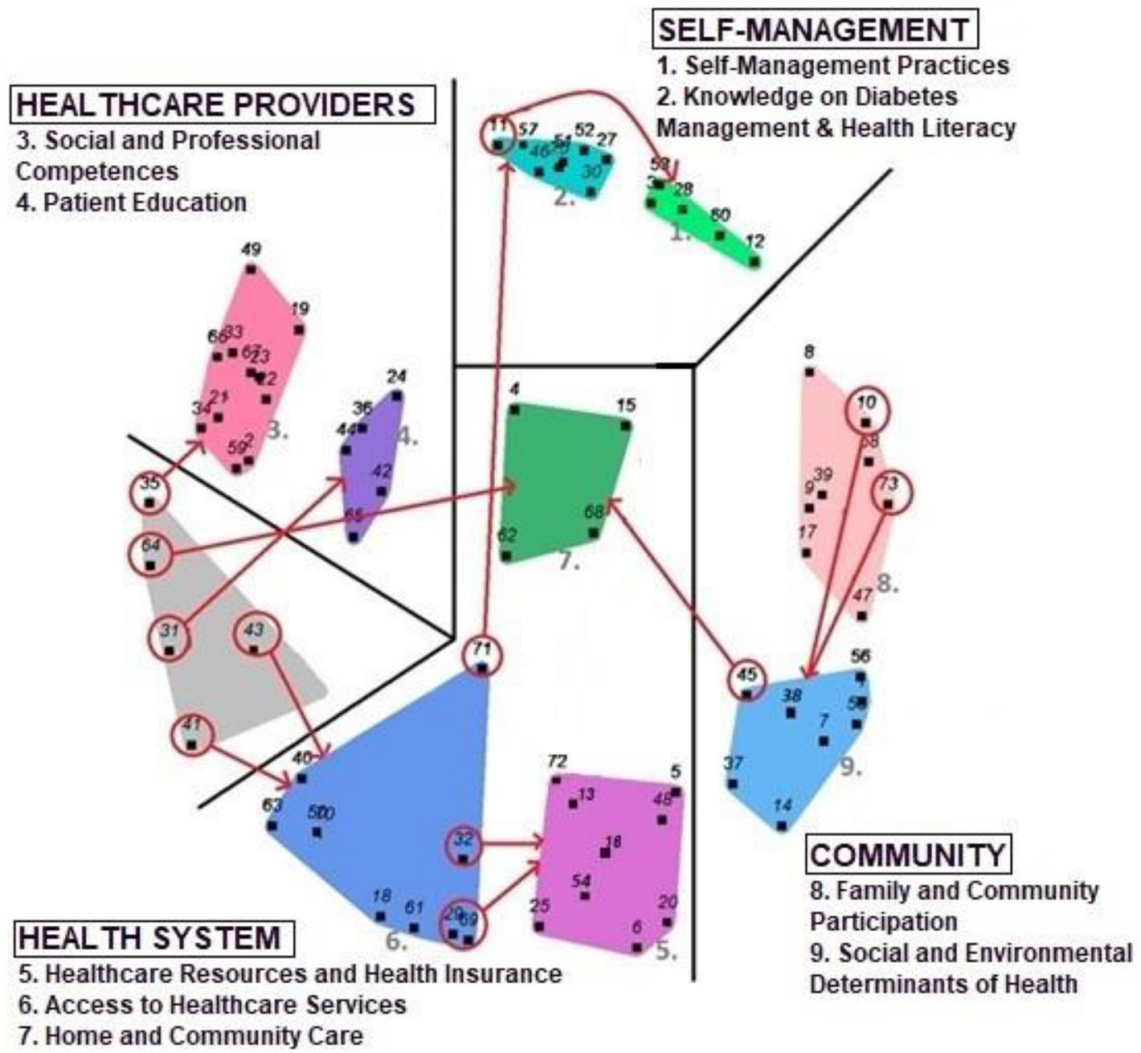
The concept map with 72 conceptually different statements represented by small black numbers, nine clusters indicated by larger white numbers and four action domains named with capital letters. Statements in a red circle are seeming anomalies that were moved to another cluster

In the original methodology participants are asked to read the statements within the computed clusters individually and form a phrase or word to describe these clusters until obtaining a group consensus on cluster names, removing erroneous clustered statements, identifying relations between clusters and grouping clusters into meaningful domains [43]. In this research, the participants did not participate in this step due to low literacy levels. The four previously mentioned persons who participated in the sorting process, named the computed clusters, moved erroneous clustered statements and identified meaningful domains. The result was the concept map as illustrated in Fig. 2.

## Results

### Socio-demographic data

Thirty-three individuals with T2D from three peri-urban municipalities of the city of Cochabamba participated in this study. Participants were primarily women (76%), over 50 years old (84%) and had not completed secondary education (67%). Most of the participants worked in the informal sector (42%), were retired (18%) or were dedicated to domestic work (34%). Many participants (61%) used natural remedies such as herbs and plants beside their prescribed medications. Only one of the 14 people who generated statements and two of the 19 people who rated the statements had a non-indigenous background, which was derived from their ability to speak an indigenous language. These and other socio-demographic data are shown in Table 1.

**Table 1:** Socio-demographic data participants

	*n (%)*	Sacaba	Quillacollo	Punata
	33	14	16	3
Sex				
Male	8 (24%)	6	2	0
Female	25 (76%)	8	14	3
Age				
41–60 years	17 (52%)	6	9	2
61–80 years	16 (48%)	8	7	1
Spoken language				
Spanish	33	14	16	3
Quechua	29	13	14	2
Aymara	2	1	1	0
Max. level of education				
No education	9 (27%)	4	5	0
Primary education	13 (40%)	6	6	1
Secondary education	9 (27%)	4	5	0
College/ university	2 (6%)	0	0	2
Occupation				
Working in informal sector	14 (42%)	9	5	0
Working in formal sector	2 (6%)	0	1	1
Domestic work	11 (34%)	1	9	1
Retired	6 (18%)	4	1	1
Pharmacological therapy				
Oral glucose lowering drugs	11 (33%)	10	0	1
Oral glucose lowering drugs + insulin	8 (24%)	2	5	1
Insulin	12 (36%)	0	11	1
None	2 (6%)	2	0	0
Use of natural remedies (herbs, plants)	20 (61%)	8	10	2
Number of years diagnosed				
1–10	13 (39%)	9	2	2
> 10	20 (61%)	5	14	1
Living situation				
Alone	4 (12%)	2	2	0
With spouse/family	29 (88%)	12	14	3

Footnote: *n* = absolute number.

### Identified domains and clustered structuring needs of people with T2D

In total, participants generated 156 original statements that were summarized in 72 conceptually different needs. These needs were rated by the participants and sorted afterwards, resulting in a concept map with 10 different clusters as shown in Fig. 2. Revising the clusters, some statements were moved to more appropriate clusters, improving their coherence, resulting in the complete redistribution of the 10th cluster. The remaining nine clusters were grouped in four overarching domains. The four domains with their underlying clusters and corresponding importance- and presence-scores are shown in Table 2. Rating scores are shown with an accuracy of two decimals, while in the written results, ratings were rounded to the closest whole number, like described earlier in both Likert scales. A 3.33 on importance for example, was rounded to 3, seen as ‘important’, while a 3.66 was considered 4 or ‘very important’. In the results, the different clusters are indicated by a number between brackets, which is also the cluster number on the concept map.

**Table 2:** Domains and clusters to achieve people-centred care for type 2 diabetes in Cochabamba, Bolivia

	Importance	Presence
**1. SELF-MANAGEMENT (14)**		
1. **Self-Management Practices (6)**	**4.55**	**3.76**
Consuming plants, herbs and other natural remedies	4.58	4.03
Maintaining a register for daily follow up of foods, symptoms, blood sugar …	4.32	2.84
2. **Knowledge on Diabetes Management & Health literacy (8)**	**4.74**	**3.23**
Having the possibility to measure the sugar level periodically	4.91	3.22
Knowing the different types of medication and their side-effects	4.61	2.96
**2. HEALTHCARE PROVIDERS (18)**		
3. **Social and Professional Competences (12)**	**4.72**	**2.71**
Healthcare personnel reduces fear of the condition through explaining the condition (psychological assistance)	4.78	2.27
A physician who knows the medical and social history of the patient	4.68	3.20
4. **Patient Education (6)**	**4.80**	**3.00**
Healthcare personnel teaching the patient about diabetes	4.76	2.81
Having information and orientation on healthy foods during medical attention	4.86	2.83
**3. HEALTH SYSTEM (25)**		
5. **Healthcare Resources and Health Insurance (11)**	**4.70**	**2.82**
Having hospitals close by that accept you when you need urgent medical care	4.94	3.29
Having enough physicians and medical material in the region	4.87	2.87
6. **Access to healthcare services (8)**	**4.77**	**2.45**
Waiting little time in line for medical assistance in healthcare facilities	4.87	1.97
Having access to consultations with a nutritionist	4.68	1.47
7. **Home and community care (6)**	**4.20**	**1.83**
Community and/ or home-visits of healthcare personnel	4.66	2.13
Having first aid or a physician on duty in the community	4.88	1.69
**4. COMMUNITY (15)**		
8. **Family and community participation (6)**	**4.70**	**2.07**
A community council engaged in enhancing health of its’ inhabitants	4.69	2.03
People with knowledge of good nutrition and food in the community who teach how to eat in a healthy way	4.69	1.53
9. **Social and Environmental determinants of Health (9)**	**4.90**	**2.85**
Having good provision of healthy foods in the neighbourhood	4.94	2.88
Having streets without dogs so you can walk and exercise in a safe way	4.75	1.88

Four domains containing nine clusters that group 72 needs for people centred diabetes care. For each cluster two needs are presented.

The number between brackets refers to the number of statements in each domain or cluster. The Bold numbers are the means of all the statements in each cluster.

### Self-management

The needs related to self-management were grouped in two clusters: ‘Self-management Practices’ and ‘Knowledge of Diabetes Management & Health literacy’.

The self-management practices (1) used in the community were perceived as essential (Importance-average = 4.55) to live with T2D on cluster-level. The statements with the highest importance and presence scores were ‘having faith in God or religion in general’ (Importance = 5.00; presence = 4.85) and ‘taking indigenous plants, herbs and other natural remedies’ (Importance = 4.58; presence = 4.03).

The second cluster on Knowledge of Diabetes Management & Health literacy (2), was considered as essential (Importance-average = 4.74) but only sometimes present (Presence-average = 3.23). Knowledge of glucose-levels (Presence = 3.19) and different types of medication and their side-effects (Presence = 2.96) was particularly low. Remarkable was the generalized lack of knowledge on how to access the healthcare system and obtain free services and medication (Presence = 1.91).

### Healthcare providers

The clusters related to the Healthcare providers were ‘Social and Professional Competences’ and ‘Patient Education’.

Social and professional competences (3) of healthcare providers were rated on cluster-level as essential (Importance-average = 4.74), yet only sometimes present (Presence-average = 2.84), like the need to trust the health provider (Importance = 4.40; Presence = 3.36). Healthcare providers failed in reducing fear and distress (Presence = 2.27) and were barely considered to be aware of family-problems (Presence = 1.63). Furthermore, health providers’ capacity to communicate in the local indigenous language was highly appreciated, yet often absent (Presence = 2.67). Great importance was attached to monthly check-ups, yet, these only occasionally took place (Presence = 3.15). Furthermore, participants expressed an urgent need for uniformity of diagnoses and treatment plans by different physicians for the same health problem (Importance = 5; Presence = 2.11).

Receiving education (4) from healthcare providers was rated as essential (Importance-average = 4.76), however only sometimes available (presence-average = 2.75). Healthcare providers seldom educated patients on T2D (Presence = 2.81), nor on medication use and diet (Presence = 2.50–2.83). Although highly valued, the need to educate family-members was rarely fulfilled (Presence = 2.50). It was remarkable that, although it was rated as essential, education on commonly used plants and herbs was nearly nonexistent (Presence = 1.78).

### Health system

The clusters ‘Healthcare Resources and Health insurance’, ‘Access to healthcare services’ and ‘Home and community care’ were grouped under the domain health system.

Aspects related to healthcare resources and health insurance (5) were rated as essential (Importance-average = 4.70), but only occasionally present (Presence-average = 2.82). Participants experienced a shortage of physicians (Presence = 2.87), medical supplies (Presence = 2.87) and medications such as insulin (Presence = 2.67). Affordable transportation to healthcare facilities was highly valued and generally available (presence = 3.88), but transportation for urgent medical assistance, such as an ambulances was practically unavailable (presence = 1.84). Health insurance was rated as very important (Importance = 4.32), however rarely perceived as available (Presence = 1.66).

The cluster, access to healthcare services (6), was rated as essential (Importance-average = 4.77), however needs were mostly unfulfilled (Presence-average = 2.45). Participants experienced long waiting times (Presence = 1.97) and a lack of guidance by their general practitioner in finding access to specialist care (Presence = 2.30). Moreover, even though perceived as essential, availability (Presence = 2.96) and affordability (Presence = 2.38) of specialist care in-hospital was perceived as deficient. Access to urgent medical care was particularly lacking (Presence = 2.23).

Home and community care (7) were rated as very important (Importance-average = 4.20) though inadequate (Presence-average = 1.83). A first aid post or a physician on duty (Importance = 4.69; Presence = 2.30) and community or home-visits by healthcare professionals were highly appreciated but practically unavailable (Importance = 4.66; Presence = 2.13). Paramedical care such as physiotherapy (Presence = 1.28) and social services (Presence = 1.26) were desired, but non-existent in the community.

### Community

The importance of community was described in two clusters: ‘Family and community participation’ and ‘Social and Environmental Determinants of Health’.

Family and community participation (8) were rated as essential (Importance-average = 4.70) but barely present (Presence-average = 2.07). Experienced support by fellow community members (presence = 2.06) and engagement of the community council in enhancing people’s health was very weak (Presence = 2.03). Furthermore, activities and gatherings for the elderly in the community (Presence = 1.63) and periodic meetings with people with diabetes and their families (Presence = 1.59) were requested but unavailable.

Needs within the cluster social and environmental determinants of health (9) were rated as essential (Importance-average = 4.90), yet only sometimes fulfilled (Presence-average = 2.85). On the one hand, most participants indicated having an adequate supply of basic utilities such as water and electricity (Presence = 4.41). On the other hand, availability of asphalted well-lit streets (presence = 2.91) and healthy foods in the neighbourhood was poor (Presence = 2.88). There was an urgent need for security in the community (presence = 2.28), which was impeded partially due to the presence of stray dogs.

## Discussion

The main objective of this study was to identify what people with T2D in Cochabamba, Bolivia needed to maintain or improve their health, and what resources they relied on, in order to develop contextualised people-centred diabetes care.

A broad range of needs was identified, but many needs remained unmet. Consistent with previous research focused on training of healthcare providers [12–15] or patient education and counselling [14, 17–22], expertise of healthcare providers at the primary health care level seemed inadequate, leaving patients with unfulfilled educational needs. However, support by healthcare professionals is pivotal in diabetes self-management education [26], leading to improved HbA1c-levels [46, 47], self-efficacy and empowerment [48], healthy coping [49] and reduced diabetes-related distress [47]. Moreover, self-management education was impeded due to poor communication skills of healthcare providers through both linguistic problems, with failure to communicate in the indigenous language [50], and the lack of a trustful continuous relationship. The latter has been shown in literature to be a condition for effective communication and interpersonal care [51] that facilitates health promotion [52–54] and metabolic control [55, 56]. A patient factor making health education challenging was low literacy levels. Less than one out of three participants finished secondary education urging for a contextualised strategy to improve health literacy which is essential for access to health care, glycemic control, prevention of retinopathy, and self-perceived health [57, 58].

In March 2019, the ‘Sistema Unico de Salud’ was introduced, a universal health system intended to provide free basic health care including periodic controls of glucose-levels, education on diet and exercise in PHC, pharmacological treatment with glibenclamide and metformin and yearly check-ups with an ophthalmologist [59]. Before this health care reform, free basic health care was limited to people over 60 [60]. Despite the prior and current universal health system, perceived access to basic health services [54] including physicians, medical supplies and essential medications was lacking. This presumably generates care seeking in the private healthcare system, putting a strain on household budgets due to higher out-of-pocket expenditures [61]. Reasons for this flee to private health care in previous studies, as well as reflected in this study were better access, shorter waiting times, better confidentiality, distrust in government institutions and better accordance to people’s needs in general [62].

Another identified need was the lack of support by fellow-community members despite the positive effect of community-support on diabetes management [63, 64]. Although a Bolivian law obliges community participation in local healthcare design [59], community members and local authorities were perceived as insufficiently engaged in health care. As such, opportunities for the community and healthcare team to co-create relevant actions on health and its determinants were missed [65]. Beside these participative needs, several needs were mentioned regarding the physical living environment, and confirmed by existing literature such as the impact of green and recreational space [66, 67], traffic noise [68, 69] and neighbourhood safety. Certain aspects such as the lack of healthy foods and the presence of stray dogs, impairing the possibility to exercise, were prominently mentioned. People-centred, as well as community oriented PHC can, with their focus on social and physical health determinants, serve as a model to consider the environment and include community members in healthcare design [65, 70].

Beside needs, resources were identified by the participants. Support was perceived both by family and religion. The latter has also proven effects on glucose-levels, coping and self-management [71, 72]. Most participants had knowledge on the use of plants and herbs with potential of improving diabetes management such as ginseng [73], ginger [74] and *Aloe vera*. *Aloe vera* has shown to reduce blood glucose levels, decrease blood lipids and promote healing of wounds such as venous ulcers [75]. Furthermore, lesser known plants were used by the study population, such as yacon, a root originated from the Andes known for its’ hypoglycemic properties, confirmed in several clinical trials [76, 77]. Reliance on these plants and herbs is interwoven with indigenous culture and must be better articulated with the Bolivian healthcare system that enacted a law on inclusion of traditional medicine [78, 79]. This articulation can promote collaborative care, wherein expertise of patients and expertise of healthcare providers is combined [23], a type of care that was not reflected in this study. Additionally, understanding people’s resources is needed for the design of an effective self-management plan in line with existing practices, knowledge and literacy levels [60, 80–82].

This research adds to an increasing body of evidence on needs and experiences of people with T2D [83–87], of which most studies were performed in HICs. Results of this research coincided partially with previous research such as the need for support from healthcare professionals [83, 85–94] and the role of family and social support in diabetes management [83, 85–87, 89]. Other results varied from previous research. First, contextualised information and education on T2D was almost absent and urgently needed [95, 96]. Causative factors for this lack of education could be the substandard training of health providers on T2D at the primary health care level and the heavy burden to educate people with low (health) literacy from different socio-cultural backgrounds in a low-resource setting. Second, many experienced distrust in healthcare institutions, resulting in non-adherence to therapies or lifestyle advice and poor self-management [97, 98]. Western medicine is frequently felt as imposed, and poorly culturally embedded [99]. Inclusion of traditionally widely used plants and herbs in health care, and communication in the local indigenous languages could promote trust. Third, the lack of supplies and medications such as insulin was experienced, while in HICs these are readily available [100].

This study led to a comprehensive exploration of needs of people living with T2D in a LMIC, which is unseen in previous studies. Because of the very broad and positively formulated seeding question: ‘Thinking as broadly as possible, what is needed to maintain or improve your health or the health of other people with type 2 diabetes in your community?’, many health needs were identified such as ‘having faith in God or religion’, going further than individual needs mentioned in the doctor’s office. As such, wider community needs have been identified, which is pivotal for planning and evaluation of health services. Health care planning based on these results goes further than traditional planning based on ratios of non communicable diseases by understanding the roots of health problems [101]. The WHO (2001) affirmed the necessity of this community health needs assessment in the planning and delivery of effective care, ensuring fair allocation of scarce resources [102].

It is important to note that this research had shortcomings. Because of the time constraints, the sample size of this study was relatively small and convenience sampling was used. This hindered in making analyses based on, for example, socio-economic status, gender, language, cultural characteristics, insurance scheme and time of diagnose; for example, women (75%) were overrepresented in this research although experiences of men and women with T2D are shown to be different [101]. While the indigenous population was overrepresented due to the recruitment in the public health system, the recruitment during an outreach project that assists deprived populations**,** and possibly due to a higher diabetes prevalence in indigenous populations in the areas under study, the perspective of people who only speak local indigenous languages was not explored. Although the modulation of the original concept methodology enhanced the generation of statements, it impeded group discussions that could have changed the structuring and interpretation of the concept map. The structure of this concept map, Fig. 2, is based on the relations the professionals perceived between the different statements; for example, the statement “having the possibility to measure the sugar level in your blood periodically” was sorted with other statements seemingly related to self-management. Notwithstanding participants could sort it together with needs related to access to health services. Another example is the statement “waiting little time in line for medical assistance in healthcare facilities”. It was sorted together more often with items related to the health system, while the participants could sort it together with items related to their health care provider. A different structure would lead to a different interpretation of the results and different actions, like discussing the problem of the waiting time with their health care providers. The interpretation of the concept map, even in the current structure, would also differ. The clusters would have been given less technical names, better understood by the community, and clusters could have been assigned to other action domains. Cluster 7, home and community care, for example is situated in the middle of the concept map. The four professionals decided that this cluster was more related to the action domain health system, while participants could relate it to the domain healthcare providers or the domain community, leading to actions like requesting training of community members by their healthcare providers. If a concept map is structured and interpreted by the participants who generated the statements, they will not only fully understand their own concept map but also take ownership, facilitating their participation in the planning and evaluation of the resulting program [103]. In future studies, support of an experienced sociologist or anthropologist and translator is desirable to facilitate inclusion of participants in all steps of concept mapping methodology, including people that only speak indigenous languages. Additionally, the inclusion of all relevant stakeholders is recommended in order to get a comprehensive understanding of what is needed for optimal diabetes management in Bolivia, and LMICs in general.

**Fig. 3:**
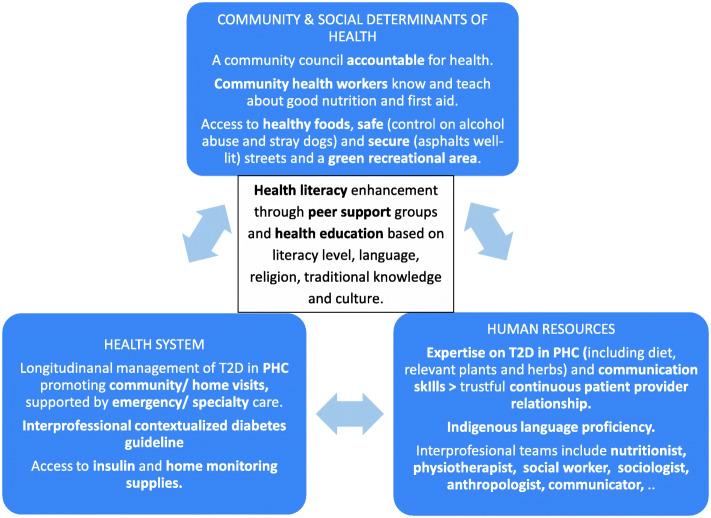
People-centred diabetes care model for recently urbanized regions in Cochabamba. *Interdependent interventions at the level of the community, the health system and human resources based on the identified needs and resources in this study are presented. T2D* *= Type 2 diabetes; PHC = Primary health care*

Findings from this research, illustrated in Fig. 3, show that the central element for a people-centred diabetes care model is reaching common ground and partnership between health care providers and the community, supported by sound health policies and health supplies, as a foundation to enhance individual and community health literacy [104]. While in Bolivia adequate health policy [37, 78] is in place, a diabetes guideline and medical supplies at the PHC level are lacking. Their presence could motivate primary health care providers to advance their knowledge and skills to manage T2D and build community partnership. As illustrated in previous research, nurses can play a crucial role in this partnership [101] and the role of community health workers must be explored [13]. Other disciplines like nutritionists, physiotherapists and social workers, as indicated by the participants, are also needed for a comprehensive needs-based health care. Although, to bridge the historical divide between biomedical medicine and traditional (indigenous) medicine as well as the traditional paternalistic health care provider patient relationship [105], professionals of other disciplines like sociologists, anthropologists and communicators are also desirable. The shift towards a primary health care led diabetes care model supported by an inter professional team and a better understanding of commonly used plants and herbs is instrumental in the design of people-centered health care and health education for people living with T2D in recently urbanized regions of Cochabamba and probably beyond.

## Conclusion

This exploratory study revealed important needs and resources of people living with T2D in peri-urban Cochabamba, Bolivia, like the urgent need to acquire knowledge and capacities to manage this condition. These needs were hampered by low literacy levels and the lack of continuous qualitative support by healthcare professionals prepared to deal with T2D in a socio-cultural and linguistic-sensitive way. Modern medicine was not always accepted due to a lack of articulation with people’s prior experiences, worldviews and commonly used natural remedies. In addition, essential health care, including medical supplies and medications, were often unavailable. The results of this study grant an opportunity to include the voice of people with T2D in the design of an inclusive, efficient and acceptable diabetes program including community-based health promotion and support activities. Taking people’s needs, resources, health literacy levels and social and cultural influences into account is essential to make diabetes care relevant and people-centred. Integrated interventions in all the proposed action domains are needed for the development of a comprehensive approach to diabetes care, wherein caregiver, care-receivers and their community become partners in improving the health of their community. Beyond the bounds of this research the results encourage critical thinking and debate on health care organisation in general.

## Data Availability

The datasets used and/or analyzed during the current study can be made available from the corresponding author on reasonable request.

## Abbreviations

T2D: Type 2 diabetes
LMICs: Low- and middle-income countries
HICs: High income countries
PHC: Primary health care
WHO: World Health Organization
